# Engagement of community stakeholders to develop a framework to guide research dissemination to communities

**DOI:** 10.1111/hex.13076

**Published:** 2020-05-25

**Authors:** Jennifer Cunningham‐Erves, Tilicia Mayo‐Gamble, Yolanda Vaughn, Jim Hawk, Mike Helms, Claudia Barajas, Yvonne Joosten

**Affiliations:** ^1^ Department of Internal Medicine Meharry Medical College Nashville TN USA; ^2^ Department of Health Policy and Community Health Georgia Southern University Statesboro GA USA; ^3^ Neighbor 2 Neighbor Nashville TN USA; ^4^ Bridges for the Deaf and Hard of Hearing Nashville TN USA; ^5^ Vanderbilt Ingram Cancer Center Vanderbilt University Nashville TN USA; ^6^ Vanderbilt University Medical Center Nashville TN USA

**Keywords:** communication, ethics, evidence‐based practice, information (research) dissemination, patient participation, research, stakeholder participation

## Abstract

**Background:**

Dissemination of research findings to past study participants and the community‐at‐large is important. Yet, a standardized process for research dissemination is needed to report results to the community.

**Objective:**

We developed a framework and strategies to guide community‐academic partnerships in community‐targeted, dissemination efforts.

**Methods:**

From 2017 to 2019, a community‐academic partnership was formed in Nashville, Tennessee, and iteratively developed a framework and strategies for research dissemination using cognitive interviews. A deductive, constant comparative analysis was conducted on interview responses to examine framework and strategy content. Feedback was used to finalize the framework and strategies for the evaluation. Using existing data, the framework's utility was evaluated in seven town hall meetings (n = 117). Bivariate analyses determined its effect on community members’ trust and willingness to participate in research using pre‐ and post‐surveys. Evaluation results were used to finalize the framework.

**Results:**

The *Community‐Engaged Research Dissemination* (CERD) framework has two phases. Phase one is a preliminary planning phase with two steps, and phase two is the four‐step dissemination process. There are five standards to be upheld conducting these phases. We provide competencies for each component. Three feasible, culturally adapted strategies were developed as exemplars to disseminate research findings. Using pre‐ and post‐surveys for intervention evaluation, there was a significant difference in trust in medical research and researchers (*P* = .006) and willingness to participate in research (*P* = .013).

**Discussion and Conclusion:**

The CERD framework can potentially standardize the process and compare the effect of dissemination efforts on the community's trust and willingness to participate in research.

## 1 INTRODUCTION

Dissemination of research findings in translational research is necessary to facilitate uptake and adoption of interventions to improve health outcomes.^1^ It involves the distribution of research findings to key stakeholders (e.g., providers, research participants, community‐based organizations [CBOs] and community members), maximizing reach and benefit of findings for target communities.^2^ However, traditional approaches to dissemination (e.g., academic publications) have limited reach and utility by patients, their families, and the community‐at‐large.^3^, ^4^ Schroter et al. (2019) found that only 27% of researchers of clinical trials disseminated results to the participants.^5^ In a study reporting dissemination in community‐based participatory research (CBPR), only 23% of researchers disseminated results to the public and 26% disseminated results to community participants at each stage of the research process (Chen et al 2010).^6^ Lack of communication about study results to these groups, particularly minority communities, strains the community‐academic partnership^7^ and contributes to mistrust in medical research(ers) and the health‐care system.^8^ Subsequently, this affects CBOs’ and community members’ understanding and willingness to engage in research opportunities.^9^, ^10^ This delays the identification of new evidence‐based knowledge, questions the generalizability of findings, and lessens use of research to improve health outcomes.^11^

Stakeholders on multiple levels increasingly recognize the value in research dissemination to study participants and the community‐at‐large. Funders (eg Patient‐Centered Outcomes Research Institute, National Institutes of Health and Agency for Healthcare Research Quality) now consistently request dissemination plans for the broader community and may request engagement of stakeholders in plan development to increase the use of findings.^12^, ^13^, ^14^ Recognizing an ethical obligation to provide findings to the community,^14^, ^15^ researchers—particularly those engaged in CBPR—are increasingly looking for effective ways to do this.^14^, ^16^, ^17^ Last, community members advocate for ‘community‐friendly’ approaches to research dissemination.^18^, ^19^, ^20^ While these strategies extend dissemination efforts to inform communities,^14^, ^16^, ^20^ there is a need for a standardized, community‐guided process for researchers across the translational research continuum to return research results to study participants and the community‐at‐large.^6^

Many conceptual frameworks exist for dissemination relating to intervention applicability in health‐care practice at community and clinical levels (i.e., dissemination and implementation science).^21^, ^22^, ^23^ For example, Harris et al (2012) developed the Health Promotion Research Center Dissemination framework to promote uptake of evidence‐based practices among user organization(s).^24^ In this model, researchers collaborate with a CBO to refine the practice and approach to dissemination using the principles of social marketing. Adoption, implementation and maintenance are steps taken by disseminating organizations for a successful outcome of change in organizational practice and personal behaviours that lead to increased productivity and improved health. These frameworks offer important concepts on dissemination, and some further demonstrate the importance of stakeholder engagement in this process.^16^, ^25^ However, a practical framework is needed that provides a stepwise, community‐guided approach to return individual research findings directly to research participants and the community‐at‐large.^6^ This is important, as few studies report providing findings to communities throughout the research process.^18^, ^26^, ^27^ Additionally, studies engaging community partners may vary in application of CBPR principles in dissemination efforts.^6^ Developing a guide for researchers to return results to the community could: (a) expand our understanding of processes to determine the findings to be disseminated; (b) identify methods to develop dissemination strategies; (c) understand the relevance and importance of the dissemination process and methods to community; and (d) potentially impact trust, willingness and participation^2^ as it relates to medical research.

The objective of this paper is to describe the development and evaluation of a practical framework and strategies to guide community‐academic partnerships in dissemination efforts with CBOs and community members participating in all phases of research. Specifically, we describe the development of a novel, community‐driven framework that was used to provide research evidence to past research participants and the community‐at‐large using community‐engaged research (CEnR) principles. This research identified the needs, priorities, and recommendations for underrepresented groups to participate in research.^18^ This work was conducted through a community‐academic partnership (one academic partner and two community organizations).

## 2 METHODS

To develop an effective, systematic process to improve academic‐community partners’ engagement in research dissemination to communities, we: (a) conducted a literature review to identify best practices and current frameworks; (b) developed an initial framework; (c) elicited feedback on the framework by conducting cognitive interviews with researchers, community leaders and members; (d) evaluated the framework, and (e) finalized the framework using study results. Collectively, we had expertise in clinical and translational research, community engagement, research dissemination and qualitative data analysis. This work was approved by the Institutional Review Boards of Meharry Medical College and Vanderbilt University.

### 2.1 Phase I literature review

We conducted a systematic literature review applying a ‘purposive’ search and article selection to gather dissemination concepts.^28^ This is not the traditional approach of exhausting the literature on the topic.^29^ We conducted the review in PubMed, CINAHL and PsycINFO from September 2018 to May 2019 using key words ‘dissemination’, ‘implementation’, ‘conceptual framework’, ‘research results’, ‘engagement’, ‘evidence‐based’ and ‘partnership’. The 6177 selected articles identified guiding principles, recommendations, models, frameworks and interventions within the context of research dissemination to communities. Using these criteria, we identified a subset of 66 articles in which we cite a few examples here.^20^, ^22^, ^23^ We completed the search once no new characteristics of dissemination emerged (i.e. saturation).

### 2.2 Phase II development of initial research dissemination framework

We used the articles to identify key concepts of research dissemination. Specifically, we coded the articles and then linked the codes using a deductive‐inductive approach. Then, we developed an initial framework depicting the process for dissemination to research participants and community‐at‐large. We further refined the framework based on our research experiences and community partner feedback.

### 2.3 Phase III stakeholder review of initial framework

We purposefully recruited two leaders from our community partners, two Latino community leaders, 12 community members and 10 researchers to evaluate the initial framework. Recruitment sites were CBOs (e.g., community health centres, colleges and churches) representative of original study participants and the community‐at‐large. Eligibility criteria included: (a) being over 18 and (b) had received or would like to receive research findings. One of our community partners was unavailable to participate at the time. However, we incorporated their feedback from the initial planning phase in framework development and while developing this paper. Eligibility criteria for researchers included: (a) having a doctoral degree (e.g., MD, PhD and DrPH), (b) conducts formative or summative research, (c) has or plans to disseminate research findings to the community and (d) receipt of external grant funding. We chose researchers with grant funding because past studies indicate they are more likely to disseminate results if expected by funders.^30^

An experienced research team member (i.e., researcher or graduate assistant) conducted the cognitive interviews by phone or in‐person. After obtaining informed consent, participants completed a brief, demographic survey. All participants answered questions on attitudes and experiences with research dissemination efforts, ways to improve efforts, and feedback (i.e., comprehension, accuracy and relevance) on the framework. Researchers were asked additional questions in the interview about research dissemination and the role their institution and community partners play in their ability to participate. Specifically, they were asked questions on barriers to research dissemination, resources/changes needed to engage in research dissemination at their institution, and needs and expectations of community partners to engage in research dissemination. We only used the feedback on comprehension, accuracy and relevance of the framework from the researchers and community members to revise the framework. The additional information collected will be reported elsewhere. Upon completion, researchers were compensated a $75 gift card for their 75‐minute interview and community members a $25 gift card for their 20‐minute interview.

Trained in qualitative data collection and analysis, members of our partnership (i.e., two researchers and a graduate assistant) transcribed the interview data. Using a line‐by‐line coding technique, we coded the text with the a priori codes ‘keep’, ‘remove’, ‘add’ or ‘clarify’ as defined in the codebook. Using a deductive approach, we used a constant comparison analysis to iteratively compare codes to determine which steps and its process in the research dissemination framework should be kept, removed, added or clarified. For both community and researcher participants, we identified all characteristics of dissemination and improved cultural appropriateness using peripheral, evidential and linguistic strategies (i.e., saturation).^31^ Participants who agreed to follow‐up were contacted for final comments, a qualitative verification procedure known as member checking.^32^

### 2.4 Phase IV evaluation of the framework's utility

To evaluate the proposed framework, we used a previously published^18^ study to provide the research findings to study participants and the community‐at‐large. Using 11 community listening sessions, the study identified the research concerns and barriers to research participation among underrepresented populations (i.e., low socio‐economic status, African American, Latino, deaf and hard of hearing). We postulated that disseminating research findings using the targeted, dissemination strategies described in the framework would increase trust in research and willingness to participate in research opportunities.

#### 2.4.1 Development and targeting of the dissemination strategies for framework evaluation

In order to disseminate research findings to past study participants and their communities, we iteratively develop three, culturally‐targeted strategies: a one‐pager, videos , and town hall meetings. Nine additional participants (three from each community partner and three from the Latino community) were purposefully recruited for 60‐minute cognitive interviews to determine cultural appropriateness of the strategies using constituent‐involving, socio‐cultural, linguistic, evidential and peripheral strategies.^31^ These participants were in the original study or were members of communities underrepresented in research. We asked participants about content, design, concerns and suggested revisions for each strategy. They were compensated with a $25 gift card. Interviews were audio‐recorded. After being transcribed, members of our partnership (i.e., two researchers) coded the data line by line. They then analysed the data using a deductive, constant comparative approach to edit the dissemination strategies iteratively. This approach was comparable to that used in framework development. We finalized the strategies.

#### 2.4.2 Data collection

We implemented seven town hall meetings throughout Nashville, Tennessee, to disseminate the research findings. We invited past study participants who had not participated in previous interviews and community members‐at‐large to participate. Our goal was 20 participants per town hall. We recruited using word of mouth, CBOs and flyers. On the day of the town hall meeting, participants viewed the PowerPoint presentation, video, and one‐pager. We provided pre‐ and post‐surveys to evaluate these strategies collectively. Measures on the pre‐survey were as follows: (a) a validated, 12‐item scale on trust in medical research and medical researchers using a 5‐point Likert scale based on agreement (Cronbach's alpha = 0.84)^33^; (b) one item on past research participation with response options yes, no, and unsure; (c) two items on willingness to participate in medical research with response options, yes, no and unsure; and (d) eight items on demographics (i.e., age, ethnicity, race, employment status, household size, gender, marital status and education). Post‐survey items only included: (a) 12‐item scale on trust in medical research and medical researchers^33^; (b) two items on willingness to participate in research; and (c) six items rating the event (e.g., *What did you like most about the event? What did you like least about the event?)*.

#### 2.4.3 Statistical analyses

We used version 23 of the IBM Statistical Package for the Social Sciences (SPSS) to analyse the data. Frequencies identified participant demographics. The chi‐squared test examined the bivariate association between willingness to participate in research pre‐ and post‐town hall. A t test evaluated the bivariate association between trust in medical research and researchers pre‐ and post‐town hall. We set the alpha level at *P* < .05.

### 2.5 Phase V framework finalization

We combined text excerpts from each post‐survey and analysed the data using the codebook and comparable data analysis used in framework and strategy development. Using post‐survey data and our experience, we finalized the content and structure of the framework. Specifically, we merged the information into the phases, steps and processes of the framework on research dissemination.

## 3 RESULTS

### 3.1 Initial framework

Using the results of our literature search and experiences of the researchers, CBO leaders and community members, we iteratively created the initial framework. First, we identified and combined similar concepts related to the return of findings to the community. Then, we identified initial steps for research dissemination and then mapped the concepts to those steps. Using those concepts, we created processes for the steps within the dissemination process. (See Supplementary File A for initial framework.) Example literature includes Harris et al (2012), Corbie‐Smith et al (2018), Brownson et al (2018), Tabak et al (2012) and Bodison et al (2015).

### 3.2 Cognitive interviews: framework development

Table 1 provides characteristics of community members, CBO leaders and researchers. All participants agreed the framework was necessary and included main components for research dissemination. Positive feedback from both researchers and community included having community involvement and the ability to adapt to all stakeholders’ needs; however, they differed in distinct ways. For example, a community member emphasized the importance of tailoring the dissemination strategy to meet their needs, while a researcher recommended adding ethics as a core value in the framework. Table 2 describes the interview results of researchers and the community along with how their input was integrated into the framework.

**TABLE 1:** Characteristics of CM/CBO and researcher interview participants for framework development, n = 26

CM/CBO (n = 16)	n (%)	Researchers (n = 10)	n (%)
Race		Race	
Caucasian	1 (6.2)	Caucasian	3 (30.0)
African American	7 (43.8)	African American	7 (70.0)
Hispanic	8 (50.0)	Hispanic	0 (0.0)
Gender		Gender	
Male	3 (18.8)	Male	2 (20.0)
Female	13 (81.2)	Female	8 (80.0)
Age		Age	
20‐30	1 (6.2)	20‐30	0 (0.0)
31‐40	3 (18.8)	31‐40	5 (50.0)
41‐50	5 (31.3)	41‐50	5 (50.0)
51‐60	5 (31.3)	51‐60	0 (0.0)
61‐70	1 (6.2)	61‐70	0 (0.0)
70‐80	1 (6.2)	71‐80	0 (0.0)
Marital Status		Number of years conducting research	
Single or Never Married	3 (18.8)	0‐5	0 (0.0)
Married	10 (62.5)	6‐10	4 (40.0)
Divorced	2 (12.5)	11‐15	2 (20.0)
Separated	0 (0.0)	16‐20	3 (30.0)
Widowed	1 (6.2)	21‐25	0 (0.0)
Education		25+	1 (14.3)
Some High School	3 (18.8)	Receipt of Grant Funding (eg NIH, PCORI and CDC)	
GED or High School Diploma	2 (12.5)	Yes	10 (100.0)
Associates Degree	1 (6.2)	No	0 (0.0)
Some College	3 (18.8)		
Bachelor's Degree	4 (25.0)		
Master's Degree	3 (18.8)		
Doctoral Degree/Professional Degree	0 (0.0)		
Medical Insurance		Dissemination of Research Findings to Community After a Study or Plan to	
Health Insurance Through employment	8 (50.0)	Yes, I have	9 (100.0)
Medicaid	0 (0.0)	Yes, I intend to	1 (0.0)
TennCare	1 (6.2)	No	0 (0.0)
Private Health Insurance	2 (12.5)		
None	4 (25.0)		
Others	1 (6.3)		
Household Income			
Less than $20 000	1 (6.2)		
$20 001‐$40 000	5 (31.3)		
$40 001‐$60 000	3 (18.8)		
$60 001‐$80 000	2 (12.5)		
Over $80 000	5 (31.3)		

Abbreviations: CBO, community‐based organization; CM, community member.

**TABLE 2:** Stakeholder engagement in framework and strategy development for research dissemination

Framework	Integrated community and researcher input
	Community Added dissemination facilitator to team and included their training in preliminary planning Changed terms in 4‐step process; defined dissemination in figure footnote Changed flow and numbering in figure Added tailoring as optional in strategy development Researcher Added information on goal development Added 5 standards for dissemination Added partnership principles Clarified role of dissemination team, community members and past research participants

Strategy	Integrated Community Input^a^
One‐Pager	● Changed colour scheme and increased font size ● Changed and centred the logo; rearranged sections ● Added term ‘deaf and hard of hearing’ ● Simplified language ● Identified errors and fixed mechanics ● Created flyer in Spanish ● Changed logo with a catchphrase
Video	● Created Spanish version ● Researchers altered author statement to show post‐study next steps ● Community partner and CM did video together; researcher was in a separate video
Town Hall Meeting	● Identified ASL interpreter for town hall ● Made programme fluid so facilitators can determine format ● Identified Spanish‐speaking facilitator ● Translated materials into Spanish (ie PowerPoint, flyers and evaluation tools)

^a^ Researcher input was not sought on the strategies as strategies chosen could vary by the dissemination objective, disseminated results and end‐users.

### 3.3 Cognitive interviews: dissemination strategy development for framework evaluation

There was an even distribution among race [Caucasian (n = 3); African American (n = 3); and Hispanic (n = 3)]; however, the majority (67%) were female. The mean age was 47. Cognitive interview participants suggested modifying content of one‐pager, video, and town hall meeting agenda to increase cultural appropriateness. Based on the data, we made changes to each strategy (see Table 2). There were no suggestions made to change the framework after showing the final framework to five participants who agreed to review post‐interview and could be reached via the contact information provided. Below is a brief description of the strategies.

*One‐Pager*. The document highlighted the partnership, the community listening sessions and study findings. The title was *1‐ACCORD*: Aspiring to Connect Communities and Researchers through Dissemination. It was available in English and Spanish.

*Targeted Videos*. We developed three five‐minute videos to disseminate results, in English, Spanish,and American Sign Language. The videos included a community partner and past research participant as spokespeople, while a researcher discussed the importance of research participation.

*Town Hall Meeting*. The agenda for town hall meetings was to describe the completed research study, show the targeted videos from the community and researcher, and provide next steps post‐dissemination. The one‐pager was distributed during the town hall, and it provided information on the study and its results. Last, a panel consisting of the academic partner, community partner(s), and a past study participant described their research experience and answered questions.

### 3.4 Evaluation results

Response rates for the seven town hall meetings ranged from 11 to 21, engaging a total of 117 community members. Majority of participants were African American (52.0%), followed by White (40.2%) and others (7.8%). Over one‐third reported being Hispanic/Latino (38.6%), married (36.8%) and male (32.4%). Majority had a bachelor's degree or lower (90.4%) and employed full time (58.2%). The average age was 48, and household size was 3. Post‐intervention, bivariate results indicated there was a significant difference in trust in medical research and researchers (*t*(205) = −2.86, *P* = .006) and willingness to participate in research (χ2(2) = 8.62, *P* = .013; see Tables 3 and 4). Table 3 describes participants’ willingness to take part in research pre‐ and post‐town halls of dissemination of research findings. Table 4 demonstrates participants trust in medical research and medical researchers pre‐ and post‐town halls of dissemination of research findings.

**TABLE 3:** Chi‐square of willingness to participate in research pre‐ and post‐town halls of research dissemination

Willingness to participate in a clinical trial	Pre (N = 112) n (%)	Post (N = 115) n (%)	*P*‐value
No	42 (37.5)	33 (28.7)	.013*
Not Sure	14 (12.5)	5 (4.3)	
Yes	56 (50.0)	77 (67.0)	

* *P* < .05.

**TABLE 4:** *t* test of trust in medical research and medical researchers pre‐ and post‐town halls of research dissemination

	Intervention						95% CI for Mean Difference	*t*	*df*
	Pre			Post					
	M	SD	n	M	SD	N			
Trust	48.4	6.33	101	51.0	6.90	106	−4.44, −0.82	−2.86**	205

** *P*<.001

Almost all participants rated the town hall meetings (98.1%) and the speakers (99.0%) as ‘good’, ‘very good’ or ‘excellent’. Text excerpts demonstrated participants liked the culturally‐targeted strategies used. Example statements were ‘I liked all the strategies’, ‘the tailoring of the presentation’ and ‘the interpreter’. Using these results, the partners added strategies to culturally target the dissemination strategies and to engage community in the dissemination process.

### 3.5 Final dissemination framework

**FIGURE 1:**
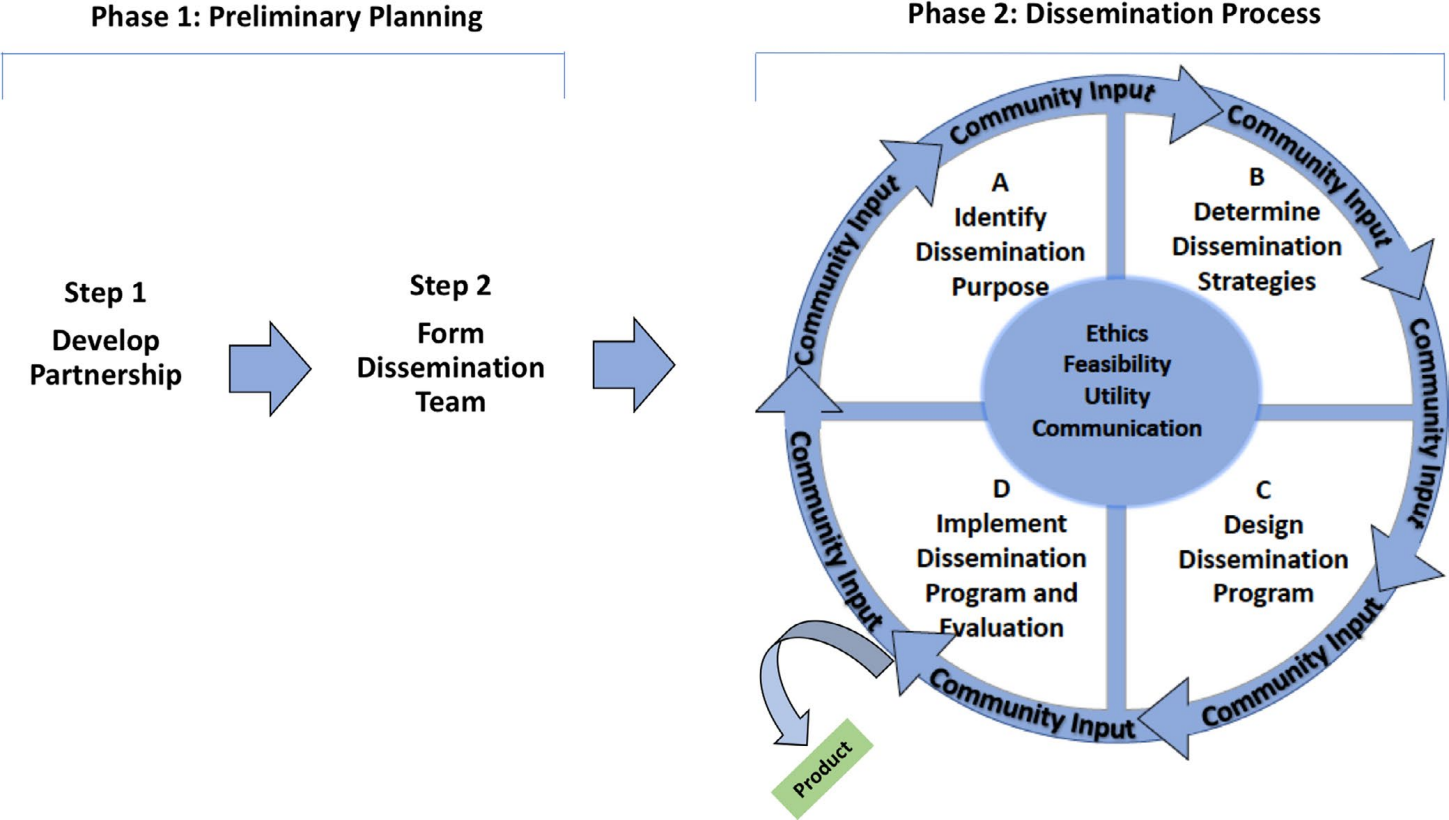
Community Engaged Research Disseminatino (CERD) Framework for Research Findings

The final Community‐Engaged Research Dissemination (CERD) framework is a two‐phase process for disseminating research in the community where phase one involves planning for dissemination effort and phase two involves conducting the dissemination process (see Figure 1). In phase one, step one involves the development of an academic‐community partnership and step two is the development of a dissemination team from members of each partnering organization to conduct phase two. There are four steps in the dissemination process of phase two: identify the dissemination purpose, determine dissemination strategies, design dissemination programme, and implement dissemination programme and evaluation with a by‐product of a dissemination tangible. Within each phase, the steps are interdependent and occur in a circular sequence. Within each step, a process occurs iteratively and has been finalized before moving to the next step. The team can move fluidly across steps in each phase if necessary. We also identify five standards to determine the quality of the dissemination effort(s): (a) utility; (b) feasibility; (c) ethics; (d) communication; and (e) ommunity Input. These standards serve as indicators of successful implementation of strategies. Table 5 describes the phases and the steps and competencies associated with each step. It also describes the standards and indicators for each standard, along with competencies to uphold each standard. See Supplementary File B for a description of the process for each step and the standards for research dissemination.

**TABLE 5:** Phases, steps and standards to disseminate study results to community using the Community‐Engaged Research Dissemination (CERD) framework

Phase 1: Preliminary planning for dissemination			
Steps	Description of step	Process at Each Step	Competencies for performing pre‐dissemination effort
1. Develop partnership	Partners that align with dissemination goals are chosen to address the dissemination effort.	Identify and select partners Identify dissemination interests Structure partnership (roles, staffing, governance, time) Develop shared learning environment and commitments Develop budget/secure funding *Note: Partnership may be existing or should be created*.	Identifies partners and stakeholders as needed Determines partners’ dissemination and research interests Establishes partner roles and responsibilities (e.g.,develop memorandum of understanding) Identifies time commitment and schedule Identifies resources of partners and community Creates a platform for shared discussion and decision making Develops a grant proposal(s) (IA)
2. Form dissemination team	After appropriate partners are selected, the dissemination team is developed and steps are taken to ensure all team members are competent in the process.	Select team from academic and CBOs Identify community members to provide input as advisors or team members Build (or continue) relationship that balances power and fosters respect and communication Foster teamwork and trust (e.g., flexibility, adaptability, honesty, and humility) Build team capacity	Identifies dissemination team members Identifies aims for the team Selects community members to advise team Sets ground rules and goals for the team Follows process for shared decision making Promotes capacity building among partner(s) as needed for the dissemination process Continues applying partnership principles

Phase 2: Dissemination process for study results to community			
Steps	Description of steps for dissemination	Process at Each Step	Competencies for performing dissemination effort
1. Identify dissemination purpose	This describes the intent of the programme to assist in evaluating the dissemination process.	Develop dissemination objective Review research findings Get community feedback and repeat steps if needed *Note: The dissemination objective should be mutually beneficial for each partner*.	Identifies aim(s) of dissemination effort Obtains team input on research findings and its’ relationship to specific aim Obtains community input on research findings and dissemination aim
2. Determine dissemination strategies	This describes the process of gauging the best strategies for research dissemination.	Identify dissemination intervention (IA) Identify strategy(s) for dissemination Finalize target population Evaluate costs and benefits of options Get community feedback on intervention(s) and option(s) and repeat process if necessary	Selects possible interventions (IA) Selects possible strategies Identifies target audience Selects process for cost/benefit analysis Skilled in gaining community feedback on intervention and/or strategy options
3. Design dissemination programme	This describes the process of programme execution and how its value is determined.	Create dissemination intervention (IA) Select and create dissemination method Create evaluation tools Get community feedback Tailor materials (optional) Get community feedback (optional) Pilot test Refine	Selects intervention development process (IA) Identifies steps for strategy development Seeks input on dissemination setting Selects recruitment procedures Identifies and trains research team members Selects methods for consent process Selects processes for data collection Identifies, selects or develops evaluation tool (IA) Identifies tests for data analysis (IA) Determines process for community feedback Identifies steps for pilot testing (IA) Selects process to tailor materials (IA) Identifies steps for refinement
4. Implement dissemination programme and evaluation	This describes the process of executing the dissemination plan and concurrent or subsequent evaluation while incorporating community input.	Implement plan Evaluate dissemination intervention Get community feedback on project/process Make refinements and repeat process if needed	Identifies successful indicators of implementation Conducts evaluation procedures Determines process for community feedback Identifies procedures for refinement

Standards for research dissemination			
Standards	Description of dissemination standard	Indicators for dissemination standard	Competencies to uphold dissemination standard
Bi‐directional communication	Communication refers to the exchange of information among community and academic partners.	Effective On‐going Shared Open Honest Bi‐directional	Determines communication process between: ‐Dissemination team members ‐Participants and team members for recruitment, strategy/programme implementation and evaluation Selects communication style Determines effectiveness of communication
Ethics	Ethics refers to conducting the dissemination process in a way that is acceptable and protects the welfare of the community.	Respect for persons Beneficence Justice	Conducts informed consent process Selects process promoting good to participants Selects steps to protect participants from harm Follows principles of Good Clinical Practice (IA)
Feasibility	Feasibility ensures the dissemination process is practical.	Environmental analysis of team approval Analysis of implementation capability	Identifies process evaluation for team Determines implementation capability
Utility	Utility ensures the dissemination process meets the needs of all stakeholders.	Culturally appropriate Readability/comprehensibility Accurate Effective Engaging	Identifies a culturally appropriate process Selects tests to determine readability Identifies procedures to determine accuracy Selects process to determine effectiveness Determines indicators for engagement
Community input	This means the community (ie patients, members) is engaged in entire process.	Serve as advisors to the dissemination team Serve as advisors throughout the process	Identifies role of a team advisor throughout the process

Abbreviations: CBO, community‐based organization; IA, if applicable.

## 4 DISCUSSION

Researchers lack effective, evidence‐based processes for research dissemination beyond traditional academic methods to ‘end‐users’. In response, we provide one of the first frameworks and strategies for the return of results to past research participants and the community‐at‐large. Our evaluation results demonstrate that this framework could be successful in increasing participants’ trust in medical researchers and the research process along with their willingness to participate in research. The *CERD* framework guides researchers and community partners to engage in a collaborative process and builds their capacity to conduct research dissemination. CEnR and partnership principles are incorporated throughout each step. To ensure an effective process, we provide five standards to guide planning and implementation of the dissemination effort. Our framework has components which overlap with existing frameworks for community engagement, CBPR, partnership development, and dissemination and implementation.^16^, ^22^ However, we define a framework that addresses an important gap to expand conceptualization of dissemination from academics to the community. This increases research relevance while meeting the needs of the community, including past research participants. Furthermore, this promotes the readiness of researchers to engage in this process while reaching dissemination goals at the national level (i.e., goals set by funders such as NIH or CDC).^34^

### 4.1 Practical application of the framework

There are implications at the individual, interpersonal, community, organizational, policy and methodological levels when conducting CEnR dissemination. Dissemination strategies may vary at each level during framework application. Using this framework at the individual level is the most fundamental with the greatest potential for an individual to engage in a behaviour change to improve public health outcomes.^35^ Engaging the community as partners in dissemination efforts can influence knowledge, attitudes, and self‐efficacy regarding research, and ultimately their participation.

Multiple levels of influence are often needed to enhance effectiveness of dissemination efforts. Studies indicate interpersonal relationships can influence an individuals’ attitude and/or behaviour.^35^ Therefore, informal groups may influence one's views and participation towards research and/or health behaviours once they engage in research dissemination. At community, organizational, and societal levels, commitment and political controversy may increase with each level.^35^ Changes are hardest at these levels, yet have the potential to produce the greatest results. At community and organizational levels, these groups can: (a) partner with academicians to conduct dissemination efforts guided by this framework and (b) promote policy changes in CBOs and community‐at‐large based on dissemination effects. Last, the result of dissemination efforts can influence policies for the funders, health‐care system, and CBOs. Thus, dissemination efforts should take into consideration the characteristics of policymakers.

Methodologically, application of CEnR and partnership principles at each level using this framework is essential. This ensures that dissemination efforts meet all stakeholders’ needs.^36^ Hence, dissemination strategies, implementation procedures, and evaluation methods and tools are effective to inform evidence‐based practices and policies for dissemination. Over time, dissemination efforts at each level can promote systemic and sustained changes in research participation and preventive behaviours, resulting in improved health outcomes.

### 4.2 Limitations

This framework was developed using community member and researcher feedback. Insight on additional stakeholder (e.g., policy makers, grant funders) perspectives came solely from the literature. Furthermore, representation of community and researchers could have been more diverse. However, this was an initial framework developed using CEnR and partnership principles to describe a dissemination process. Also, there was only one researcher who intended to disseminate results; however, she shared similar perspectives related to the dissemination process. Future studies should explore ways for sample representativeness for framework evolution. Next, while this framework was developed based on one dissemination project, (a) we gauged the views of researchers who had engaged in community research dissemination and community members who would like to receive or have received study findings, and (b) our implementation process appeared to result in an increase in willingness to participate in clinical research and trust in medical research and researchers on the part of community members. Future studies are needed to confirm these results.

## 5 CONCLUSIONS

Researchers can apply the *CERD* framework to partner with communities and implement the proposed strategies to disseminate research findings. The framework: (a) guides researchers to engage in community research dissemination; (b) ensures community engagement principles in all phases of planning and implementing dissemination; (c) ensures ethical guidelines are met; (d) increases value and usability of research findings for community members and CBOs; and (e) ensures all stakeholder needs are met for evaluation. Ultimately, framework application could improve implementation of research dissemination efforts, creating a foundation for robust dissemination efforts. Implementing this process could build rapport and trust in communities, particularly those underrepresented in research, influencing research participation and public health outcomes.

## CONFLICT OF INTEREST

None reported.

## ETHICAL APPROVAL

Ethical approval has been granted exemption by Meharry Medical College Institutional Review Board (Protocol #18‐07‐844) for interviews conducted with researchers and community stakeholders to better understand the dissemination process while applying principles of community engagement.

## Data Availability

Research data are not shared due to ethical restrictions.
