# Supplementary figures and images for: Engagement of community stakeholders to develop a framework to guide research dissemination to communities
Source: Health Expect. 2020 May 25;23(4):958–68. doi: 10.1111/hex.13076 (PMC7495063; doi:10.1111/hex.13076)

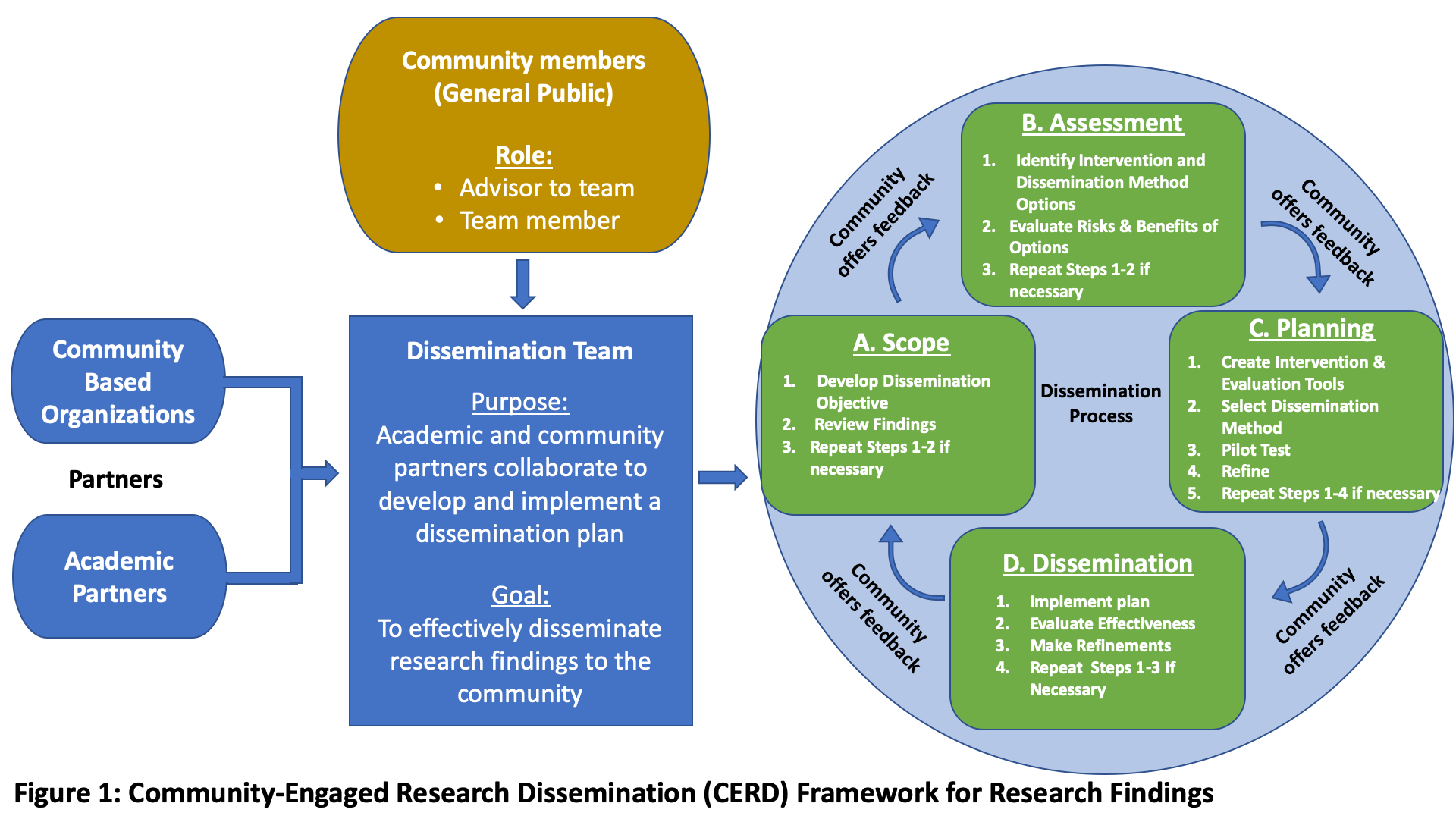

Supplement: Supplementary file 1 [file HEX-23-958-s001.tiff]
