# Supplementary material for: Engagement of community stakeholders to develop a framework to guide research dissemination to communities
Source: Health Expect. 2020 May 25;23(4):958–68. doi: 10.1111/hex.13076 (PMC7495063; doi:10.1111/hex.13076)
Supplement: Supplementary file 2 [file HEX-23-958-s002.docx]

**Supplementary File B**

Description of 2-Phase Dissemination Process using the Community-Engaged Research Dissemination (CERD) Framework

*Phase 1: Preliminary Planning*

*Develop Partnership.* The academic-community partnership is the core element for the dissemination process. Principles of CEnR and partnership development provide the foundation for trust and ensures mutual benefit. These principles include creating a memorandum of understanding, establishing governance and ground rules, determining infrastructure, promoting respect and conflict resolution, balancing power, practicing cultural humility, and identifying appropriate funding distribution.^1^ The partnership should be established prior to planning the dissemination effort, ideally at the beginning of the research process.^2, 3^ There are two primary partners-academic and community. However, partners may be added/or removed throughout the process. Each partner identifies their short and long-term dissemination interests, ensuring transformation can occur at multiple levels (e.g., personal, institutional, or community transformation) for goal development.^3^ Excluding partners in initial planning phases could cause dissemination efforts to be stalled, ignored, criticized, or even resisted.

*Form Dissemination Team.* Members of CBOs, academic institutions, and community-at-large (e.g., patients, caregivers, clients) form the dissemination team. Community members may serve as advisors or team members. The individual(s) responsible for disseminating the strategy or intervention should be on the team. Degree of involvement of each team member could vary based on research dissemination activities and partner preference. However, the aim is for all partners to be engaged throughout the process. CEnR and partnership principles should continue to be applied to foster trust, teamwork, capacity building, and sustainability.^2, 3^

*Phase 2: Conduct Dissemination Process*

After the partnership and dissemination team are developed, the process can begin. There are four steps: (1) Identify Dissemination Purpose; (2) Determine Dissemination Strategies; (3) Design Dissemination Program; and (4) Implement Dissemination Program and Evaluation.

*Identify Dissemination Purpose.* The dissemination effort must be targeted to address needs of partners and overall community while efficiently using time and resources. A dissemination objective is developed to create unity among partners and reach the study’s overarching goals. To further develop the objective, the team should review the research findings for dissemination. Researcher and community members may have different ideas about which findings are important, and how to interpret them. Therefore, it is important to follow partnership principles in setting the goal for the dissemination program. Once set, community input on the goal is sought, using a CEnR approach. This process may be repeated to ensure the dissemination effort is relevant and mutually beneficial.

*Determine Dissemination Strategies.* To determine the best strategy(ies), the dissemination goal and literature should be reviewed and stakeholders’ views (e.g., targeted community) considered. Examples of how this input can be solicited are interviews, community advisory boards, focus groups, and community engagement studios.^4^ Reach and utility of each strategy should be considered to optimize community acceptance. Strategies for research dissemination include, but are not limited to community-partnered conferences, mailings, phone or in-person meetings, traditional media (e.g., newspaper, radio, TV), podcasts, social media, CBOs, and websites.^5, 6^ These strategies can be incorporated into interventions to assess their effectiveness. Interventions typically occur on the intrapersonal level. However, interventions on interpersonal, community/organizational, or societal levels should be considered.^7^ Potential intervention types are education, communication, policy, community mobilization, and behavioral interventions. Partners should rank two or three strategies for consideration, and conduct rankings for interventions if applicable.

Cost-benefit analyses of the strategies or interventions can help determine the best choice for research dissemination. Available resources (i.e., money, time, personnel, space), appropriate fit for users, and projected effects on behavioral outcomes should be evaluated in the analyses.^8^ If the selected strategies have costs that outweigh benefits, this step should be repeated. After completing the assessment, partners should choose the best strategy to fit their needs.

*Design Dissemination Program.* After a strategy is chosen, the team should design their dissemination program. All plans need action steps, program facilitators, a communication plan, and a timeline. For the chosen strategy(ies) or intervention, a brief synopsis of the research results, how they will be used, and identification of researchers who conducted the research should be included. Each strategy should also provide contact information for the researcher and community partner if community members have subsequent questions. The strategy could also be adapted using CEnR, partnership, and social marketing principles.^2, 3^ While optional, targeting the strategy(ies) or intervention should occur to increase cultural appropriateness to participants. Targeting can be achieved through personalization, feedback, and content aligning using evidential, linguistic, socio-cultural, peripheral, and constituent-involving strategies.^9^ Once developed, strategies can be refined based on community feedback.

If these strategies are tested in an intervention, evaluation materials should be developed to determine if dissemination goals and objectives were met. Evaluation procedures include focus groups, surveys, interviews, and analytics. A pilot test should be conducted among the target population. The intervention could be finalized or refined depending on the implementation process, strategy effectiveness, and participant evaluations. If refinement is necessary, the team determines if step three is repeated.

*Implement Dissemination Program and Evaluation*. Implementing the dissemination plan is the most important step. The plan may be as simple as mailing a one-pager or as rigorous as implementing and evaluating a research dissemination program. Prior to implementation, facilitators or staff to implement the dissemination plan should be trained. For implementation, communication models and standards (e.g., clarity, content, channels) are available to ensure the strategy(ies) are effective.^10^ If the strategy is tested in an intervention, the method and/or materials, or the effect of dissemination on an outcome (e.g., attitudes toward research, engagement in future research or preventive behaviors) is evaluated. This will be done using the strategies identified in the *Design Dissemination Phase*. The evaluation process should engage all stakeholders, be focused, and gather credible information. Upon completion, the results of the evaluation should be returned to the stakeholders.

*Standards for Dissemination*

We identified five standards to determine the quality of dissemination efforts: 1) Utility; 2) Feasibility: 3) Ethics; 4) Communication; and 5) Community Input. They can serve as indicators of successful implementation of strategies.

*Ethics.* During the research dissemination process, CBOs and community members must be respected, protected, and rights are met.^11^ They should be fully informed and understand the process, including associated risks and benefits. If the dissemination strategy is tested in an intervention, informed consent should be taken. The strategies/intervention, along with associated benefits and costs, should be equitably distributed across participants. Upon study completion, the outcomes and next steps should be provided to the community comprehensively and timely.^12^

*Feasibility.* The dissemination process must be practical. The level of acceptance of the dissemination effort is identified across partners. If differences exist, they should be addressed. Implementation capacity is evaluated early to prevent delay. This involves assessing adaptive, leadership, management, and operational capacities of the partners.^13^ It determines if the space, skills, time, and funds are available to conduct the process. Not all researchers will have the funding to support active dissemination strategies.

*Utility.* Dissemination strategies should meet all stakeholder needs. Stakeholders, including the intended end-user, should be identified prior to initiating the process to ensure effectiveness, cultural appropriateness, comprehensibility, and accuracy.

*Bi-directional Communication.* Bi-directional communication is required in both phases of dissemination. It is transactional and occurs across various mediums. Stakeholders, including community organizations and patient representatives, should be encouraged to be open and honest about their needs and concerns when sharing their perspectives on research dissemination.

*Community Input.* Community input from past research participants and community-at-large is essential to develop dissemination strategies or interventions which meet the needs of the targeted audience. They provide perspectives on the research dissemination topic and/or the proposed dissemination process, potentially preventing implementation of ineffective dissemination strategies. They may serve as an advisor to the dissemination team or advisors at each step in the dissemination process.

**References**

1. Michener L, Cook J, Ahmed SM, Yonas MA, Coyne-Beasley T, Aguilar-Gaxiola S. Aligning the goals of community-engaged research: why and how academic health centers can successfully engage with communities to improve health. *Academic medicine.* 2012;87(3):285-291. doi: 10.1097/ACM.0b013e3182441680

2. Clinical and Translational Science Awards Consortium Community Engagement Key Function Committee Task Force on the Principles of Community Engagement. Principles of Community Engagement. Washington, D.C.: U.S. Government Printing Office; 2011.

3. Israel BA, Eng E, Schulz AJ, Parker EA. *Methods in Community-Based Participatory Research for Health.* 2nd Edition ed. San Francisco, CA: Josey-Bass; 2012.

4. Joosten Y, Israel T, Head A, et al. Community engagement studios: A structured approach to obtaining meaningful input form stakeholders to inform research. *Academic Medicine.* 2015;90(12):1646-1650. doi: 10.1017/cts.2018.323

5. Brownson RC, Eyler AA, Harris JK, Moore JB, Tabak RG. Getting the Word Out: New Approaches for Disseminating Public Health Science. *Journal of public health management and practice.* 2018;24(2):102-111. doi:10.1097/PHH.0000000000000673

6. Bodison SC, Sankare I, Anaya H, et al. Engaging the Community in the Dissemination, Implementation, and Improvement of Health-Related Research. *Clinical and translational science.* 2015;8(6):814-819. doi: 10.1111/cts.12342

7. Whitehead M. A typology of actions to tackle social inequalities in health. *J Epidemiol Community Health.* 2007;61(6):473-478. doi:10.1136/jech.2005.037242

8. Centers for Disease Control and Prevention. Cost-Benefit Analysis. 2019; https://www.cdc.gov/policy/polaris/economics/cost-benefit-analysis.html. Accessed May 30, 2019.

9. Hawkins RP, Kreuter M, Resnicow K, Fishbein M, Dijkstra A. Understanding tailoring in communicating about health. *Health education research.* 2008;23(3):454-466.

doi:10.1093/her/cyn004

10. U.S. Department of Health and Human Services, National Institute of Health, National Cancer Institute. Make Health Communications Programs Work. Washington, D.C.: U.S. Department of Health and Human Services; 2004.

11. Shalowitz DI, Miller FG. Disclosing individual results of clinical research: implications of respect for participants. *Jama.* 2005;294(6):737-740. doi:10.1001/jama.294.6.737

12. Wong CA, Hernandez AF, Califf RM. Return of Research Results to Study Participants: Uncharted and Untested. *Jama.* 2018;320(5):435-436. doi:10.1001/jama.2018.7898

13. Office of Adolescent Health. Assessing Organizational Capacity. 2015; https://www.hhs.gov/ash/oah/sites/default/files/assessorgcapacity-slides.pdf. Accessed May 30, 2019.
